# Polychlorinated dioxins, furans (PCDD/Fs) and dioxin‐like polychlorinated biphenyls (dl‐PCBs) in food from Italy: Estimates of dietaryintake and assessment

**DOI:** 10.1111/1750-3841.15901

**Published:** 2021-09-08

**Authors:** Grazia Barone, Arianna Storelli, Antonio Busco, Rosanna Mallamaci, Maria M. Storelli

**Affiliations:** ^1^ Biosciences, Biotechnologies and Biopharmaceutical Department University of Bari “Aldo Moro”—Strada Prov. le per Casamassima Km 3 Valenzano BA Italy

**Keywords:** diet, dl‐PCBs, food safety, PCDD/Fs, public health

## Abstract

**Abstract:**

Dietary intake of polychlorinated dioxins and furans (PCDD/Fs) and dioxin‐like polychlorinated biphenyls (dl‐PCBs) from various foods (fish and seafood, meat and meat‐based products, milk and dairy products, hen eggs, olive oil and fats) was investigated for various sex/age groups of the Italian population. The concentrations of PCDD/Fs and dl‐PCBs and their contribution to total TEQ values varied depending on food matrix. Fish (0.50 pg WHO‐TEQ/g wet weight) and seafood (0.16 pg WHO‐TEQ/g wet weight) showed the highest mean concentrations of PCDD/Fs plus dl‐PCBs, followed by meat (1.70 pg WHO‐TEQ/g lipid weight), meat based products (1.03 pg WHO‐TEQ/g lipid weight), milk and dairy products (0.78 pg WHO‐TEQ/g lipid weight), hen eggs (0.71 pg WHO‐TEQ/g lipid weight), fats (0.27 pg WHO‐TEQ/g lipid weight) and olive oil (0.09 pg WHO‐TEQ/g lipid weight). In all samples WHO‐TEQ PCDD/F plus dl‐PCB concentrations fulfilled the European Union food law, except in pork loin samples (1.39 pg WHO‐TEQ/g lipid weight). Differences in exposure depending on the sex/age groups (children > teenagers > adults > elders) and hypotheses considered (lower bound and upper bound) were encountered. Non‐cancer risk values showed a low exposure. Carcinogenicity risk results revealed that highly exposed individuals were distributed over all sex/age groups, even though the proportion of individuals exceeding the safe limit was higher in children. These data once again underline the importance of trying to control the levels of these contaminants in fishery products, particularly in fish, who represents one of the main exposure sources for consumers.

**Practical Application:**

This paper may help the consumer in making food choices to minimize the exposure risk to dioxins, furans and PCBs

## 1 INTRODUCTION

Polychlorinated biphenyls (PCBs) and polychlorinated dibenzo‐*p*‐dioxins and dibenzofurans (PCDD/Fs) are three classes of toxic polyhalogenated aromatic hydrocarbons characterized by high chemical and metabolic persistence. Anthropogenic release of PCBs has resulted in a widespread contamination of the soils and sediments. Similarly, the release of PCDD/Fs during a variety of industrial and thermal processes as well as by a number of natural processes, such as volcanic eruptions and forest fires has pervaded the environment, generating contaminated sites and hot spots. It is broadly recognized that the major route of exposure of PCBs and PCDD/Fs to humans is through dietary uptake. Due to their lipophilic character, these chemicals tend to concentrate in lipid rich foods as meat, fatty fish, milk and dairy products inducing a wide spectrum of toxic responses including infertility, reproductive system disorders, immunological toxicity, and carcinogenic effects (WHO, 2000; SCF, 2001).

The number of dietary contamination episodes occurred during the last decades has highlighted the need to strengthen the legislative measures either to reduce the presence of these pollutants in the environment or to keep their levels within safe limits in food. The European Union (EU) has, in fact, set maximum levels permissible for PCDD/Fs and dioxin‐like‐PCBs (dl‐PCBs) in foods, expressed as toxic equivalents (WHO‐TEQ), in regulation No 1259/2011 (Official Journal of the European Union, 2011) currently in force. In a similar way, the World Health Organization (WHO) has set up provisional tolerable intakes of PCDD/Fs and dl‐PCBs, on daily basis (PTDI: 2 pg WHO‐TEQ/kg b.w.; WHO, 1998), on weekly basis (PTWI: 14 pg WHO‐TEQ/kg b.w.; (SCF, 2001) and on monthly basis (PTMI: 70 pg WHO‐TEQ/kg b.w.; JECFA, 2002). In this picture it is central to underline that European Commission asked EFSA's expert Panel to provide a scientific opinion on the risks due to the presence of dioxins (PCDD/Fs) and dioxin‐like PCBs in feed and food for human and animal health. As result, the panel of experts set a new TWI for dioxins and dioxin‐like PCBs in food of 2 pg WHO‐TEQ/Kg body weight, seven times lower than the previous TWI (EFSA, 2018). In the light of this, the continuous monitoring of the levels of these contaminants in food is needed to prevent health human risks and evaluate the trend of human exposure hence the effectiveness of the specific management measures.

The food monitoring studies on PCDD/Fs and PCBs in various European Countries have been performed in the last decades (Marin et al., 2011; Perelló et al., 2012; Quijano et al., 2018; Sirot et al., 2012; Törnkvist et al., 2011; Windal et al., 2010) until now (Hulin et al., 2020), but to the best of our knowledge, currently available data about the dietary exposure in Italian population are rather limited (Fattore et al., 2008; Fattore et al., 2006; Taioli et al., 2005; Turci et al., 2006) and often dedicated merely to a few dietary components (Barone et al., 2019; Ghidini et al., 2005; Grassi et al. 2010; Esposito et al., 2020; Bartalini et al., 2020; Castellani et al., 2021).

In this research, the levels of PCDD/Fs and dl‐PCBs were measured in composite food samples from five varieties of food groups (seafood, meat and based meat products, milk and dairy products, hen egg, olive oil and other fats) to evaluate their compliance with the maximum permissible limits (MPLs) set by European Union regulation. The dietary intake of PCDD/Fs and dl‐PCBs was subsequently estimated for age/gender subgroups of the Italian population by comparison with the new provisional tolerable weekly intake recommended by EFSA. At the end, the potential human health risks were evaluated using the hazard quotient (HQ) and lifetime cancer risk (LCR).

## 2 MATERIALS AND METHODS

### 2.1 Sample collection

In May–July 2019, food samples were randomly acquired in supermarkets representing the five most popular retailer brands in Italy. The various supermarkets were located in 6 cities (Bari, Lecce, Taranto, Foggia, Brindisi and Matera) of Southern Italy. A total of thirty‐five types of foods classified in the following groups: (1) fish (rosefish, European hake, red mullet, common sole, bluefin tuna) and seafood (cephalopods: common octopus, common cuttlefish, European squid), (shellfish: Mediterranean mussel, striped venus clam, common scallop), (crustaceans: red shrimp, spottail mantis shrimp, Norway lobster); (2) meat (veal fillet, pork loin, chicken breast, turkey breast) and meat‐based products (salami, mortadella, raw ham, baked ham); (3) milk (cow whole milk) and dairy products (hard cow cheese, hard goat cheese, yoghurt, mozzarella, stracchino, ricotta, mascarpone); (4) eggs from free foraging chickens; (5) extra virgin olive oil and fats (butter, margarine, mayonnaise) were acquired from five supermarkets of each city. For each food, four individual items were taken from each supermarket of the six cities and combined into a composite sample. For shellfish and some crustaceans (i.e., red shrimp and spottail mantis shrimp), 10 individual units were included to prepare the composite sample. For bluefin tuna, slices (*n* = 30) of about 0.1−0.2 kg of muscle tissue were taken. The composite samples (only edible part) were homogenized and stored below −20°C. For each food item, two composite samples were prepared for the analytical determination.

### 2.2 Analytical method

The concentrations of twelve “dioxin‐like” PCBs (dl‐PCBs): non‐*ortho* PCBs 77, 81, 126, 169 and mono‐*ortho* PCBs 105, 114, 118, 123, 156, 157, 167, 189) together with the seventeen 2,3,7,8‐ substituted PCDD/F congeners were determined. The analytical method has been reported in detail in previous paper (Barone et al., 2019).

For the determination of dl‐PCBs, homogenized samples (0.5−3.0 g) were mixed with Na_2_SO_4_ and spiked with PCB 143 (internal standard) and extracted with hexane. A liquid–liquid extraction with organic solvents adapted to the matrix type was applied to liquid samples (Eljarrat et al., 2002), whereas olive oil samples were directly dissolved in hexane. The eluated were evaporated to dryness under a stream of nitrogen and the lipid content was gravimetrically determined. The extracts were then eluted through an acidified silicagel column (H_2_SO_4_, 44% w/w), using 50 mL of a mixture of hexane/dichloromethane (1/1, v/v) for elution of the analytes. The eluate was evaporated to dryness and redissolved in 100 µl of iso‐octane.

For the determination of PCDD/Fs (US EPA method 1613), the samples extracted, as above reported, were subjected to a multistep clean up to remove the matrix and the potential interfering components. The first stage was a fat destruction step consisting of a treatment of the sample solution with sulphuric acid and base back‐extraction. The obtained extracts were then subjected to a preconditioned florisil clean‐up column, which was eluted with different solutions in order to remove interfering components. The first eluted solvent was discarded, while the second eluate containing PCDD/Fs was collected. The extracts were evaporated to dryness and redissolved in iso‐octane. Appropriate C^13^‐labeled extraction standards were added to the samples in order to control the whole sample preparation process. The final obtained PCBs and PCDD/Fs extracts were injected and analyzed separately.

### 2.3 Instrumental analysis

PCDD/Fs and dl‐PCBs analysis were performed on a HRGH/HRMS system consisting in a MAT 95 XL mass spectrometer, coupled with a GC Trace series 2000 (Thermo Electron, Darmstadt, Germany). Chromatographic separation was carried out with a Trace Gold TG‐Dioxin capillary column (60 m × 0.25 mm i.d. × 0.25 μn film thickness; Thermo Fisher Scientific, Waltham, MA, USA) for PCDD/Fs and with a Trace TR‐PCB8 MS capillary column (50 m × 0.25 mm i.d. × 0.25 μn film thickness; Thermo Fisher Scientific) for dl‐PCBs. Helium (99.9999% purity) at 1 ml/min was used as carrier gas and the temperatures of ion source and transfer line were set at 260 and 290°C, respectively. Injections (1 µl) were performed in split less mode on an A200S autosampler (Thermo Fisher Scientific). Electron ionization mode (E.I.), as well as voltage selected ion recording mode (VSIR) was chosen as operating method. Electron energy was 35 eV. The detector resolving power was >10.000 (10% valley definition) and the two most intense ions were monitored for the determination of the single congeners. Perfluorokerosene (PFK) was the mass reference used. The quantification was performed by isotope dilution method. Multi‐level calibration curves (*r* ^2^ > 0.999) in the linear response interval of the detector were created for the quantification. The calibration curves were prepared to result in a range of 0.5–800 ng/ml for PCB congeners and in a range of 0.025–2.00 pg/µL for PCDD/Fs.

### 2.4 Quality assurance and quality control

Quality assurance and quality control (QA/QC) was performed through the analysis of procedural blanks, quantitative control sample for each batch of samples, duplicate sample, and a standard reference material (CARP‐2 National Research Council of Canada, Ottawa, Canada; ERM–BB445 Joint Research Center, Geel, Belgium) for each set of samples. For the replicate, standard reference materials and recovery of labelled compounds (AccuStandard Inc., New Haven, USA; Wellington laboratories Inc., Guelph, ON, Canada), the relative standard deviations (RSD) were <10% for all the detected compounds. The recovery rates of labelled standards were between 85 and 120%. Obtained values were deviating with less than 20% from the consensus values. The limits of detection (LODs) were calculated as three times the signal‐to‐noise ratio and varied amongst analyte groups (0.0038−0.16 pg/g for PCDD/Fs and 0.04−1.40 pg/g for PCBs). The limits of quantification (LOQs) were the followings: 0.04−1.0 pg/g for PCDD/Fs and 0.12−3.80 ng/g for PCBs. Concentrations of PCBs and PCDD/Fs are expressed as pg WHO‐TEQ/g on wet or lipid weight basis in accordance with the EU legislation (Official Journal of the European Union, 2011).

### 2.5 Exposure assessment

The dietary intakes of PCDD/Fs plus dl‐PCBs were calculated via deterministic approach. The estimated dietary intakes were calculated by multiplying the food consumption data by mean TEQ concentrations of dioxin‐like PCBs and PCDD/Fs in each food and then dividing by the body weight. Dietary habits and biometric data of the total population and of various sex/age classes (children: 3–9.9 years, body weight 26.1 kg; male teenagers: 10–17.9, body weight 57.1 kg; female teenagers: 10–17.9, body weight 49.1 kg; male adults: 18–64.9 years, body weight 78.4 kg; female adults: 18–64.9 years, body weight 62.2 kg; male elders: ≥65 years, body weight 78.1 kg; female elders: ≥65 years, body weight 65.0 kg) were obtained by the Italian national food consumption survey INRAN‐SCAI (Leclercq et al., 2009). The concentrations of TEQs for PCDD/Fs and dioxin‐like PCBs were obtained using the World Health Organization (WHO)‐Toxic equivalency factors (TEFs) established in 2005 (Van den Berg et al., 2006). For exposure calculations, the contamination level of each sample expressed in lipid weight was converted into wet weight using the lipid content of the samples. Lower and upper bound (LB and UB) concentrations were calculated assuming that all values of the non detected congeners are equal to zero and limit of detection (LOD), respectively. Kruskal–Wallis nonparametric test was undertaken to compare the estimated weekly intake according to the gender for each age group. All *p*‐values below 0.05 were considered statistically significant. Statistical analyses were achieved using XLSTAT‐R version 2019.1 (Addinsoft, Paris, France).

Cancer and noncancer health risks were determined on the estimated dietary intake of PCDD/Fs plus dl‐PCBs. The noncancer risk evaluation was assessed on the Hazard Quotient (HQ) and was calculated by dividing the daily intake by the reference dose (RfD). The reference dose for the sum of PCDD/Fs and dl‐PCBs is 0.7 pg TEQ/kg body weight/day (US EPA, 2012). An estimated HQ over 1 is considered representing a major risk of adverse health effects. Carcinogens do not have an effective or safe threshold. For carcinogenic effects, the risk is expressed as the probability of contracting cancer over a lifetime (LCR) and was calculated by multiplying the daily intake by the cancer slope factor (CSF). Oral cancer slope factors of 1.5 × 10^−4^ pg/kg body weight/day (US EPA, 1985, 1994) and 1 × 10^−3^ pg/kg body weight/day (US EPA, 2000) were used for cancer risk calculations. This latter represents the US EPA's most current upper bound slope factor for estimating human cancer risk based on human data. LCR values greater than one in one million (1 × 10^−6^ pg/kg body weight/day) are considered unacceptable, while the US EPA (US EPA, 2000) considers a risk greater than one in one hundred thousand (1 × 10^−5^ pg/kg body weight/day) to be unacceptable.

## 3 RESULTS AND DISCUSSION

### 3.1 WHO‐TEQ contamination levels

Concentrations of PCDD/Fs and PCDD/Fs plus dl‐PCBs expressed in picograms of WHO‐TEQ are illustrated in Tables 1, 2, 3. Additionally, in the same tables, the maximum permissible limits (MPLs) for the sum of dioxins and dl‐PCBs set by European Union (Official Journal of the European Union, 2011) have been reported. In accordance with the EU legislation on food, our concentrations are expressed on a lipid basis for all of the food evaluated, except seafood for which results are expressed on a wet weight basis.

**TABLE 1:** Concentrations of PCDD/Fs, dl‐PCBs, and PCDD/Fs plus dl‐PCBs expressed as WHO‐TEQ (pg/g) and maximum permissible levels (MPLs) set by European Union regulation (Official Journal of the European Union, 2011) for fish and seafood

Fish and seafood	WHO‐PCDD/F‐TEQs	WHO‐dl‐PCB‐TEQs	WHO‐PCDD/F plus dl‐PCB‐TEQs	MPLs WHO‐PCDD/F plus dl‐PCBs‐TEQs
Rosefish	0.14	0.19	0.33	6.5 pg/g wet weight
European hake	0.04	0.14	0.18	6.5 pg/g wet weight
Red mullet	0.10	0.17	0.27	6.5 pg/g wet weight
Common sole	0.13	0.26	0.39	6.5 pg/g wet weight
Bluefin tuna	0.08	1.24	1.32	6.5 pg/g wet weight
Fish (average)	0.10	0.40	0.50	–
Common octopus	0.04	0.001	0.04	6.5 pg/g wet weight
Common cuttlefish	0.01	0.001	0.01	6.5 pg/g wet weight
European squid	0.02	0.02	0.04	6.5 pg/g wet weight
Cephalopods (average)	0.02	0.01	0.03	–
Mediterranean mussel	0.32	0.12	0.44	6.5 pg/g wet weight
Striped venus clam	0.28	0.06	0.34	6.5 pg/g wet weight
Common scallop	0.12	0.07	0.19	6.5 pg/g wet weight
Shellfish (average)	0.24	0.08	0.32	–
Red shrimp	0.08	0.01	0.09	6.5 pg/g wet weight
Spottail mantis shrimp	0.08	0.01	0.09	6.5 pg/g wet weight
Norway lobster	0.07	0.01	0.08	6.5 pg/g wet weight
Crustacean (average)	0.08	0.01	0.09	–
All seafood (average)	0.13	0.03	0.16	–

**TABLE 2:** Concentrations of PCDD/Fs, dl‐PCBs, and PCDD/Fs plus dl‐PCBs expressed as WHO‐TEQ (pg/g) and maximum permissible levels (MPLs) set by European Union regulation (Official Journal of the European Union, 2011) for meat and meat products

Meat and meat products	WHO‐PCDD/F‐TEQs	WHO‐dl‐PCB‐TEQs	WHO‐PCDD/F plus dl‐PCB‐TEQs	MPLs WHO‐PCDD/F plus dl‐PCBs‐TEQs
Veal fillet	1.35	0.20	1.55	4.0 pg/g lipid weight
Pork loin	1.15	0.24	1.39	1.25 pg/g lipid weight
Chicken breast	1.62	0.20	1.82	3.0 pg/g lipid weight
Turkey breast	1.58	0.42	2.00	3.0 pg/g lipid weight
Meat (average)	1.43	0.27	1.70	–
Salami	0.29	0.82	1.11	1.25 pg/g lipid weight
Mortadella	0.65	0.21	0.86	1.25 pg/g lipid weight
Raw ham	0.91	0.11	1.02	1.25 pg/g lipid weight
Baked ham	0.40	0.73	1.13	1.25 pg/g lipid weight
All meat products (average)	0.56	0.47	1.03	–

**TABLE 3:** Concentrations of PCDD/Fs, dl‐PCBs, and PCDD/Fs plus dl‐PCBs expressed as WHO‐TEQ (pg/g) and maximum permissible levels (MPLs) set by European Union regulation (Official Journal of the European Union, 2011) for milk, dairy products, eggs, and fats

Milk and dairy products	WHO‐PCDD/F‐TEQs	WHO‐dl‐PCB‐TEQs	WHO‐PCDD/F plus dl‐PCB‐TEQs	MPLs WHO‐PCDD/F plus dl‐PCBs‐TEQs
Milk	1.19	0.18	1.37	5.5 pg/g lipid weight
Hard cheese (cow milk)	0.37	0.71	1.08	5.5 pg/g lipid weight
Hard cheese (sheep milk)	0.48	0.72	1.20	5.5 pg/g lipid weight
Yoghurt	1.18	1.02	2.20	5.5 pg/g lipid weight
Mozzarella	0.04	0.02	0.06	5.5 pg/g lipid weight
Stracchino	0.08	0.06	0.14	5.5 pg/g lipid weight
Ricotta	0.02	0.01	0.03	5.5 pg/g lipid weight
Mascarpone	0.03	0.14	0.17	5.5 pg/g lipid weight
Milk and dairy products (average)	0.42	0.36	0.78	–
Hen eggs	0.10	0.61	0.71	5.0 pg/g lipid weight
Olive oil	0.05	0.04	0.09	1.25 pg/g lipid weight
Other fats				
Butter	0.13	0.09	0.22	1.25 pg/g lipid weight
Margarine	0.15	0.11	0.26	1.25 pg/g lipid weight
Mayonnaise	0.19	0.14	0.33	1.25 pg/g lipid weight
Fats (average)	0.16	0.11	0.27	–

As expected, fish (0.50 pg WHO‐TEQ/g wet weight) and seafood (0.16 pg WHO‐TEQ/g wet weight) was the food group showing the highest mean concentrations of PCDD/Fs plus dl‐PCBs. The other food categories were in the following order: meat (1.70 pg WHO‐TEQ/g lipid weight) and meat‐based products (1.03 pg WHO‐TEQ/g lipid weight), milk and dairy products (0.78 pg WHO‐TEQ/g lipid weight), eggs (0.71 pg WHO‐TEQ/g lipid weight), fats (0.27 pg WHO‐TEQ/g lipid weight), and olive oil (0.09 pg WHO‐TEQ/g lipid weight).

Within seafood, excluding fish, the highest degree of contamination was in shellfish (0.32 pg WHO‐TEQ/g wet weight), followed by crustaceans (0.09 pg WHO‐TEQ/g wet weight) and cephalopods (0.03 pg WHO‐TEQ/g wet weight).

For meat, a small margin between veal fillet (1.55 pg WHO‐TEQ/g lipid weight) and pork loin (1.39 pg WHO‐TEQ/g lipid weight) concentrations was observed as well as slightly higher levels were found in turkey breast (2.00 pg WHO‐TEQ/g lipid weight) compared to chicken breast (1.82 pg WHO‐TEQ/g lipid weight). Among meat‐based products, baked ham (1.13 pg WHO‐TEQ/g lipid weight) and salami (1.11 pg WHO‐TEQ/g lipid weight) appeared to be more contaminated, followed by raw ham (1.02 pg WHO‐TEQ/g lipid weight) and mortadella (0.86 pg WHO‐TEQ/g lipid weight).

Concerning milk and dairy products the highest value corresponded to yoghurt (2.20 pg WHO‐TEQ/g lipid weight), followed by milk (1.37 pg WHO‐TEQ/g lipid weight) and hard cheese samples (sheep milk: 1.20 pg WHO‐TEQ/g lipid weight; cow milk: 1.08 pg WHO‐TEQ/g lipid weight), while the remaining products exhibited a lower contamination level (0.03–0.17 pg WHO‐TEQ/g lipid weight).

For fats, a large variation was observed with olive oil samples having lower levels (0.09 pg WHO‐TEQ/g lipid weight) respect to other group components (0.22–0.33 pg WHO‐TEQ/g lipid weight). The wide concentration fluctuation either in various food categories or within the same group is obviously linked to multiple factors. For instance, the structure and the dynamic of the food webs appear to be a determinant of contamination levels in marine biota. Within fish, top predator species with longer food webs tend to have higher levels than those with lower trophic levels (Klinčić et al., 2020; Storelli et al., 2008). Similarly, the high concentration variability encountered in the other seafood analyzed is caused not only by the functional traits of organisms, but also by the combined effect of the feeding behavior and diet of each species (Garcìa et al., 2000; Storelli et al., 2007). As for meat, the lower contamination of pork respect to veal is reasonably attributable to the short economic life of fattening pigs and their fat mass leading to a dilution of these lipophilic contaminants (Malish et al., 1999). Likewise, the PCDD/F and dl‐PCB enrichment in cheeses and yoghurt respect to ricotta might be due to hydrophobic clotting of milk casein during ripening (De Filippis et al., 2013).

### 3.2 Percentage of contribution from each food group to the WHO‐TEQ contamination levels

The contribution of PCDD/Fs and dl‐PCBs to the total TEQ concentrations also varied depending on the food matrix. For fish, the percentage contribution to total WHO‐TEQ from dl‐PCBs was dominant accounting for 80.0%, unlike other seafood that exhibited a higher percentage of PCDD/Fs (cephalopods: 66.7%; shellfish: 75.0%; crustaceans: 88.9%). These findings are consistent with those found in other studies confirming that PCBs are more strongly biomagnified in food chain than PCCD/Fs (Barone et al., 2014; Fattore et al., 2006; Marin et al., 2011; Perelló et al., 2012).

In meat (82.7–89.0%) and milk (86.9%) too, the main contribution to the WHO‐TEQ came from the PCDD/Fs, whereas in eggs were more abundant dl‐PCBs (85.9%). Within meat‐based products, the contribution percentage of PCDD/Fs and dl‐PCBs to total TEQ was largely variable. Specifically, in salami (73.9%) and baked ham (64.6%) noticeable was the role of dl‐PCBs, whereas in mortadella (75.6%) and raw ham (89.2%) samples, PCDD/Fs toxicity equivalents were greater than those of dl‐PCBs.

For milk and dairy products too, the contribution of PCDD/Fs or dl‐PCBs to the cumulative TEQ was different based on the food type. Both cow and goat milk cheeses and mascarpone showed a prevalence of dl‐PCBs (60.0%−82.4%) unlike milk (86.9%) and the remaining dairy products in which a predominance of dioxins was observed (53.6%−66.6%).

In olive oil and fat group, the contributions of PCDD/Fs and dl‐PCBs to the total TEQ values were very similar and ranged between 55.5% and 59.3%. In general terms, these findings reflect the existence of different sources of contamination between foodstuffs of aquatic origin and land‐based products and confirm the robust dl‐PCBs contribution to the cumulative TEQ in fish (Marin et al., 2011; Perelló et al., 2012).

### 3.3 Comparison with literature data

An overview with literature data, although complicated due to several factors (see differences in the number of congeners tested, differences in the approach for the calculation, ways to express the contaminant concentrations, etc.), indicate that the mean concentrations of PCDD/Fs plus dl‐PCBs found here are reasonably coherent with the findings from different studies conducted in other European countries, such as Spain (meat and meat‐based products: 0.93 pg WHO‐TEQ/g lipid weight; milk and dairy products: 0.99 pg WHO‐TEQ/g lipid weight; eggs: 0.78 pg WHO‐TEQ/g lipid weight; oil and fats: 0.37 pg WHO‐TEQ/g lipid weight; Marin et al., 2011), Austria (meat and meat products: 1.16 pg WHO‐TEQ/g lipid weight; cheese: 0.83 pg WHO‐TEQ/g lipid weight; butter: 0.50 pg WHO‐TEQ/g lipid weight; Rauscher‐Gabernig et al., 2013), Belgium (fish: 0.01–1.35 pg WHO‐TEQ/g wet weight; meat and meat products; 0.21–1.78 pg WHO‐TEQ/g lipid weight; milk and dairy products: 0.53‐1.74 pg WHO‐TEQ/g lipid weight; eggs: 0.64–1.14 pg WHO‐TEQ/g lipid weight; Windal et al., 2010) and France (fish: 2.72 pg WHO‐TEQ/g wet weight; milk: 1.10 pg WHO‐TEQ/g lipid weight; Tard et al., 2007).

### 3.4 Compliance with EU regulation and exposure assessment

Although human exposure to these chemicals can occur in various ways, food is the primary source for the general population. In consequence, understanding the contaminant levels in food is a key issue for evaluating the human exposure and to prevent possible diseases. To this end, an important first step is to keep the concentrations of these toxic substances at a reasonable level to ensure the lowest possible exposure to consumer.

The European regulation No 1259/2011 (Official Journal of the European Union, 2011) sets maximum permissible limits (MPLs) for human consumption in many foods. As shown in Tables 1, 2, 3, the limits for PCDD/Fs plus dioxin‐like PCBs, expressed as TEQ value, are different for the various food categories. Following these legislative measures, all samples tested showed WHO‐TEQ PCDD/F plus dl‐PCB concentrations below the requirements of European Union food law, with the exception of pork loin samples (1.39 pg WHO‐TEQ/g lipid weight) showing levels slightly above the allowable limit.

However, to protect health, it is not enough to keep concentrations below EU maximum limits but also is necessary to set threshold values of human exposure to these harmful chemicals. As before mentioned, recently the European Food Safety Authority (EFSA) on the basis of new epidemiological and experimental data on animal, has re‐assessed the human health risk related to the presence of PCDD/Fs and dl‐PCBs in food establishing a new tolerable weekly intake (TWI) value of 2 pg WHO‐TEQ/kg body weight (EFSA, 2018).

As shown in Table 4, all exposure estimates exceeded the tolerable dietary intake, although it is clear that the results expressed as upper bound resulted in an exposure overestimation principally determined by food consumption with very low contamination levels. However, looking at the data more specifically, it was observed that in lower bound scenario the estimated weekly intake for total population was slightly higher than tolerance limit being 2.47 pg WHO‐TEQ/kg body weight/week, whereas reached 3.52 pg WHO‐TEQ/kg body weight/week in upper bound hypothesis. Children were the population group with the highest exposure of PCDD/Fs and dl‐PCBs with a value from three to four times higher (LB: 6.04 pg WHO‐TEQ/kg body weight/week, UB: 8,65 pg WHO‐TEQ/kg body weight/week) than the recommended TWI, followed by teenagers with intakes estimated from two to three times the fixed limit (LB: males: 3.49 pg WHO‐TEQ/kg body weight/week, females: 3.74 pg WHO‐TEQ/kg body weight/week; UB: males: 4.98 pg WHO‐TEQ/kg body weight/week, females: 5.25 pg WHO‐TEQ/kg body weight/week). Finally, adults (LB: males: 2.46 pg WHO‐TEQ/kg body weight/week, females: 2.66 pg WHO‐TEQ/kg body weight/week; UB: males: 3.51 pg WHO‐TEQ/kg body weight/week, females: 3.77 pg WHO‐TEQ/kg body weight/week) and elders (LB: males: 2.31 pg WHO‐TEQ/kg body weight/week, females: 2.13 pg WHO‐TEQ/kg body weight/week; UB: males: 3.43 pg WHO‐TEQ/kg body weight/week, females: 3.11 pg WHO‐TEQ/kg body weight/week) both showed intake values slightly higher than the limit threshold mentioned above in lower bound hypothesis and values almost double under upper bound scenario (Table 4).

**TABLE 4:** intake of PCDD/Fs plus dl‐PCBs (pg WHO‐TEQ kg b.w. week^−1^) via food consumption for total population and for various sex/age classes considered

**Food groups**	Total population		Children		Teenagers				Adults				Elders			
					**M**		**F**		**M**		**F**		**M**		**F**	
	LB	UB	LB	UB	LB	UB	LB	UB	LB	UB	LB	UB	LB	UB	LB	UB
Fish and seafood	1.06	1.20	2.72	3.07	1.47	1.65	1.73	1.95	1.00	1.13	1.19	1.34	1.03	1.30	0.84	0.95
Meat and meat products	0.30	0.44	0.70	1.02	0.54	0.77	0.45	0.64	0.34	0.49	0.29	0.42	0.26	0.38	0.22	0.33
Milk and dairy products	0.66	1.09	1.50	2.64	0.91	1.54	0.92	1.55	0.65	1.07	0.71	1.19	0.58	0.97	0.64	1.07
Hen eggs	0.36	0.50	0.93	1.29	0.45	0.62	0.52	0.72	0.38	0.53	0.37	0.51	0.36	0.50	0.35	0.49
Olive oil and fats	0.09	0.29	0.19	0.63	0.12	0.40	0.12	0.39	0.09	0.29	0.10	0.31	0.08	0.28	0.08	0.27
Total intake	2.47	3.52	6.04	8.65	3.49	4.98	3.74	5.25	2.46	3.51	2.66	3.77	2.31	3.43	2.13	3.11

M = male; F = female; LB = Lower Bound; UB = Upper Bound.

From these data, a decreasing trend emerged with age, with children having an intake double that of teenagers and three times that of adults and the elderly. The high value found in children should be due to the different dietary habits with the rest of the population together with their lower body weight, factors that strongly influence exposure. However teenagers were also highly exposed with a total intake of about twofold higher than in adults and elderly people. Furthermore, with regard to gender, dietary intake was higher in females teenagers and adults than males, while an opposite trend was observed in the elderly. However all intake values did not reach levels of statistical significance between genders (*p* > 0.05), probably due to nearly similar dietary patterns and amount consumed within each age group.

The relevance of the exposure data was largely determined by marine products whose consumption alone constituted a considerable percentage of the established TWI in all sex/age classes (over 40%, see next paragraph). Particularly important in this evaluation was the observation that within the fish group, bluefin tuna was the main contributor to the intake of these pollutants for all sex/age groups. In this context, excluding tuna from the exposure estimates, there were two general considerations that capture attention and that must necessarily be discussed. The first was connected to food distribution pattern, which remained almost unchanged within the different population groups considered, with the exception of children for whom the contribution of milk to the total intake equalized that of seafood. The second aspect concerned the type of fish, the frequency of consumption and the meal size which are crucial issues to adequately balance the health benefits and risks of regular fish consumption (Domingo, 2016).

This deserves even more attention in connection with the fact that potential impact of these chemicals on human changes dramatically at life stages with wider and more critical windows not only in infants and children, but also in the elderly population. In fact, the physiological changes that accompany the normal aging process result in a progressive deterioration of bodily functions and the ability to respond to environmental stress (Risher et al., 2010). Furthermore, any pathological state that compromises the function of any organ can further decrease the body's ability to protect itself, exposing the elderly population to the negative effects generated by these contaminants.

However, in assessing and characterizing the human exposure through food, uncertainties and limitations in many aspects of the data (consumption statistics, sample representativeness, etc.) and methods (presentation of concentrations as lower, medium or upper bound) need to be considered. In our particular case, to reduce the uncertainty associated with consumption data, more accurate information on the quantities consumed by the different sex/age groups for each item, within each food category considered, would have been helpful. In addition, the expression of the results with lower bound approach introduces a large margin of imprecision in estimated exposure levels. Furthermore, food preparation and/or cooking methods alter dioxin and PCB levels in the final product (Van Leeuwen et al., 2007) helping to increase the uncertainty of estimated exposure levels. If you add these and other potential types of uncertainties together, it goes without saying that intake values calculated must be interpreted with caution. However, despite these uncertainties, our data gives an overall evaluation of the exposure to these pollutants in the various age and sex groups considered.

### 3.5 Percentage of contribution from each food group to the estimated TEQ intake

In the total population the highest percentage contributor to the intake of these pollutants corresponded to seafood, 42.9% of total TEQ, followed by milk and dairy products (26.7%), hen eggs (14.6%), meat and meat products (12.1%), and olive oil and fats (3.7%).

In children, in addition to fishery products which represented 45.0% of the total intake, a significant source of PCDD/Fs and dl‐PCBs was the milk and dairy products whose intake, two to three times higher than in other age groups, accounted 24.8% of the total. Another important route of exposure included eggs (0.93 pg WHO‐TEQ/kg body weight/week), which accounted 15.4% of the total intake, followed by meat and meat‐based products constituting 11.6%, while olive oil and other fats with a contribution equal to 3.1%, played a marginal role in total exposure. As regards free‐foraging chickens eggs it should be emphasized that their consumption can be a relevant route of exposure for human (Weber et al., 2018). In fact, several studies have shown that free‐range chickens have a higher content of these pollutants than confined laying hens as consequence of the ingestion of contaminated soil, insects and worms. This leads to a bioaccumulation of pollutants in egg fat at levels often above the EU food standard Kijlstra et al., 2007; Roszko et al., 2014).

Concerning the teenagers, the contribution of dioxins and dl‐PCBs to total EWI from marine product consumption was slightly greater in females (46.3%) than males (42.1%). Milk and dairy products as well as the consumption of meat and meat‐based products made an important contribution to total intake showing a higher percentage in males (milk and dairy products: 26.1%; meat and meat‐based products: 15.5%) respect to females (milk and dairy products: 24.6%; meat and meat‐based products: 12.0%), unlike eggs whose contribution to total intake was higher in females (13.9%) compared to males (12.9%), whereas olive oil and other fats gave a percentage contribution of around 3.0% in both sexes.

Adult males and females received 40.7% and 44.7% respectively of their exposure from fish and other seafood, followed by milk and dairy products, whose consumption resulted of 26.0% for both genders. The category of meat and meat‐based products determined a higher percentage in males (13.8%) than females (10.9%), whereas consumption of olive oil and other fats set around at 4.0% in both sexes.

Finally, in oldest age group the exposure levels from marine products were higher in males than females representing 44.6% and 39.4% of the total intake, respectively. The consumption of meat and meat‐based products (males: 11.3%, females: 10.3%), eggs (males: 15.6%, females: 16.4%) and olive oil and other fats (males: 3.5%, females: 3.8%) contributed to the total intake with percentages almost similar in both sexes, whereas the females (30.0%) were more exposed respect to males (25.1%) solely via milk and dairy product consumption.

### 3.6 Human health risk

Health risk assessment is one of the best approaches to investigate the potential risk of exposure to toxic substances for human, offering relevant information to public health decision‐makers to protect the consumer health. The findings of analysis for both carcinogenic and noncarcinogenic risks relative for different sex/age groups are shown in Table 5. Regarding the noncarcinogenic risk, the calculated HQ values indicated the absence of risk associated with dietary exposure for all sex/age groups, excluding children. In this population group in fact a value slightly greater than one was observed (HQ = 1.24). Also lifetime cancer risk (LCR) in children were also above the US EPA maximum acceptable value of 1 × 10^−6^ pg/kg body weight/day, which means that one case of cancer occurs in every 1,000,000 individuals. On this basis, it was found that intake of these chemicals on daily basis via the various foods considered by children could possibly lead to the appearance of 13 cancer cases out of 100,000 individuals. A lower, although still significant, cancer risk was also found in all other sex/age groups studied with values ranging from 4.57 × 10^−5^ pg/kg body weight/day to 8.01 × 10^−5^ pg/kg body weight/day. The risk assessment results are sensitive to the choice of dose–response functions, and the selection of a function is often an important source of uncertainty in risk assessment. Consequently, a slope factor of 1 × 10^−3^ pg/kg body weight/day developed on human data basis was also used as an upper bound cancer risk estimator. On this basis, LCR values ranging from 3.04 pg/kg body weight/day to 8.63 pg/kg body weight/day were above the safe limit indicating a possibility of more cancer victims (Table 5).

**TABLE 5:** Hazard quotient (HQ) and the lifetime cancer risk (LCR) via food consumption and for various sex/age classes considered

Sex/age classes	Noncancer risk (HQ)	Cancer risk (slope factor 1.5 × 10^−4^)^a^	Cancer cases per 100,000 people	Cancer risk (slope factor 1 × 10^−3^)^b^	Cancer cases per 10,000 people
Total population	0.50	5.30 × 10^−5^	5	3.53 × 10^−4^	4
Children	1.24	1.30 × 10^−4^	13	8.63 × 10^−4^	9
Male teenagers	0.71	7.49 × 10^−5^	7	4.99 × 10^−4^	5
Female teenagers	0.76	8.01 × 10^−5^	8	5.34 × 10^−4^	5
Male adults	0.50	5.27 × 10^−5^	5	3.51 × 10^−4^	4
Female adults	0.54	5.70 × 10^−5^	6	3.80 × 10^−4^	4
Male elders	0.47	4.96 × 10^−5^	5	3.30 × 10^−4^	3
Female elders	0.43	4.57 × 10^−5^	5	3.04 × 10^−4^	3
Acceptable risks	–	≤1 × 10^−6^	–	≤ 1 × 10^−5^	–

^a^US EPA, 1985, 1994.

^b^US EPA, 2000.

## 4 CONCLUSION

PCDD/Fs and dl‐PCB levels were determined in five different food categories to estimate dietary intakes of different sex/age groups of the Italian population. Fishery products showed the highest levels, followed by milk and dairy products, meat and meat products, and to a lesser extent hen eggs and olive oil and other fats. The levels measured in the composite samples were all well below the maximum limits set by the European Commission for human consumption, with the exception of the salami samples. The estimated intake of total population was slightly above the new tolerance limit of 2 pg WHO‐TEQ/kg b.w./week. Within different age groups, children had the highest exposure followed by teenagers and by adults and elderly. Although literature data show that dietary intake of PCDD/Fs and dl‐PCBs in the population has decreased in recent decades (Gonzales et al., 2018; Tard et al., 2007), our data, particularly those relative to young consumers, exceeding the TWI of 2 pg WHO‐TEQ/kg b.w./week highlights the need to maintain efforts to reduce exposure. This consideration is also supported by the risk assessment, which has been estimated to be above the level of concern for cancer risk in all population groups considered. According to the obtained results it is recommended to continue these studies by adding other dietary products in order to monitor the levels of these harmful chemicals in order to ensure a high level of public health protection.

## AUTHOR CONTRIBUTIONS

Grazia Barone: Data curation; methodology. Arianna Storelli: Data curation; formal analysis; reviewing and editing; Maria Maddalena Storelli: methodology; validation; writing—original draft; writing—review & editing. Antonio Busco: Software. Rosanna Mallamaci: Data curation.

## CONFLICTS OF INTEREST

Authors declare to not have any conflicts of interest.
